# Exploring the feasibility of implementing the SPELL-Links to Reading and Writing intervention

**DOI:** 10.1007/s11881-024-00315-w

**Published:** 2024-08-30

**Authors:** Katlynn Dahl-Leonard, Colby Hall, Philip Capin

**Affiliations:** 1 Department of Curriculum, Instruction, and Special Education, University of Virginia, Charlottesville, VA USA; 2 Meadows Center for Preventing Educational Risk, University of Texas at Austin, Austin, TX USA; 3 Department of Curriculum and Instruction, University of Houston, Houston, TX USA; 4 Harvard Graduate School of Education, Harvard University, Cambridge, MA USA

**Keywords:** Determinants of implementation, Fidelity, Reading intervention

## Abstract

This study examined six reading intervention teachers’ implementation of the SPELL-Links to Reading and Writing intervention with students in Grades 2 and 3. The purpose was to explore the extent to which teachers who received a one-day training session without ongoing coaching support were able to implement the intervention with fidelity. It also aimed to better understand the determinants (i.e., barriers and facilitators) of teachers’ implementation and their perceptions related to the importance, feasibility, and effectiveness of the intervention. Data from four sources (implementation logs, implementation observations, interviews, and surveys) were collected and analyzed. Participating teachers did not implement the intervention at the intended dosage of 4 days per week, but they did demonstrate high adherence and quality on average. Several barriers to implementation were identified, including intervention training, intervention content and structure, compatibility with existing practices, and alignment with goals. Notable facilitators of implementation were teacher capability and peer support. Teachers had varying perceptions of the intervention, with neutral satisfaction on average. These findings indicate a need for further research on determinants of intervention implementation as they may be crucial in supporting teachers’ implementation fidelity.

**Supplementary Information:**

The online version contains supplementary material available at 10.1007/s11881-024-00315-w.

There is a profound gap between empirical findings about evidence-based reading instructional practices and typical practice in school settings (Kretlow & Helf, 2013; Solari et al., 2020). There are multiple reasons for this gap, including lack of access to science-aligned curricula and instructional materials. However, even when teachers have access to evidence-based reading programs, successful implementation of such programs can be challenging. Implementation science research has identified several factors that may influence the feasibility of implementing evidence-based reading interventions in authentic school contexts (Damschroder et al., 2022; Proctor et al., 2011), including intervention-level (e.g., program attributes, such as cost and design quality), individual-level (e.g., the knowledge or motivation of the teacher implementing the intervention), and school-level factors (e.g., the priority placed on implementing the intervention). The present study focused on the feasibility of reading intervention teachers of students in Grades 2 and 3 implementing an evidence-based literacy intervention. In particular, we evaluated the extent to which teachers are able to implement the intervention with fidelity and explored the teacher-reported intervention-level, individual-level, and systems-level determinants (i.e., barriers and facilitators) of implementation fidelity.

## Implementation fidelity

Conceptual and empirical work has indicated that implementation fidelity is a multidimensional construct (e.g., Dane & Schneider, 1998; O’Donnell, 2008). Although the conceptualization of fidelity and its core components varies across research disciplines, Dane and Schneider’s five dimensions of fidelity are one of the most comprehensive examinations of fidelity and have been applied to study implementation fidelity in early literacy interventions (Guo et al., 2016). According to Dane and Schneider, the five dimensions of fidelity are dosage, adherence, quality, responsiveness, and differentiation. *Dosage* refers to the amount of instruction provided or how much exposure to an intervention students received. *Adherence* refers to the extent to which critical components of the intervention are implemented as intended. *Quality* of instructional delivery describes qualitative aspects of implementation, such as implementer preparedness and pacing. *Responsiveness* is the extent to which students respond to the intervention. *Differentiation* is the extent to which the intervention varies from another treatment condition or a comparison condition. The present study focused on three of these dimensions: dosage, adherence, and quality.

Prior research suggests a positive association of dimensions of implementation fidelity with evidence-based instruction and academic outcomes for students (Kretlow & Bartholomew, 2010; Vadasy et al., 2015; Wolgemuth et al., 2014). For example, Wolgemuth et al. (2014) reported that intervention adherence and quality of delivery accounted for variance in phonological awareness and word reading outcomes among treated students. Vadasy et al. (2015) revealed that greater intervention dosage was associated with kindergarten students’ gains in vocabulary and decoding. They also found that greater intervention adherence was associated with student gains in vocabulary and spelling. Importantly, implementation fidelity is most strongly associated with reading outcomes for low-performing students (Boardman et al., 2016; Capin et al., 2022; Connor et al., 2007). For example, Connor et al., (2007) found that third-grade students with low initial reading performance disproportionately benefited from the high-fidelity implementation of an explicit reading intervention when compared to their higher-performing peers. Therefore, how teachers implement interventions is an important part of understanding why interventions are or are not successful for students with reading difficulties.

However, reviews of fidelity reporting within intervention research show that fidelity is frequently unreported. Swanson et al. (2011) reviewed articles published in select general and special education journals from 2005 to 2009 and found that only 47% of studies reported fidelity data. Capin et al. (2018) examined treatment fidelity in K-3 reading intervention research and found that only 47% of the reading intervention studies reported fidelity data. More recently, Dahl-Leonard et al. (2023a) examined fidelity reporting within reading intervention studies for elementary students with or at risk for dyslexia and found that 75% of studies reported fidelity data. In all three syntheses (Capin et al., 2018; Dahl-Leonard et al., 2023a; Swanson et al., 2011), the authors discovered that studies reporting fidelity data primarily reported treatment adherence, with other dimensions of fidelity often absent from the corpus of studies reviewed.

The lack of fidelity reporting within these studies is concerning because implementation fidelity is a crucial consideration for researchers aiming to make causal inferences about intervention effects and stakeholders (e.g., policy makers and school leaders) aiming to make decisions about curricula and instructional approaches. Measuring and reporting fidelity increases the internal validity of a study evaluating the intervention’s effects (i.e., when an intervention is implemented with fidelity, it is more possible to infer that the reported effects are indeed due to the intervention being evaluated; Shadish et al., 2002). Additionally, research that assesses the implementation fidelity of an intervention is vital in showing whether the intervention is feasible for teachers to implement in authentic classroom contexts. Overall, determining the feasibility of implementation is important because it informs judgments about the practicality of scaling up the intervention and generalizing to other populations and settings (Nelson et al., 2012; O’Donnell, 2008; Solari et al., 2020).

## Determinants of implementation

In addition to measuring and reporting fidelity of implementation, there is value in identifying factors that impact intervention feasibility. A commonly used implementation science framework, the Consolidated Framework for Implementation Research (CFIR; Damschroder et al., 2022), identifies characteristics that support or hinder the implementation of evidence-based practices (i.e., determinants of implementation). Notably, the CFIR describes intervention-level, teacher-level, and school-level determinants.

According to the CFIR (Damschroder et al., 2022), intervention-level determinants of implementation include the intervention source, evidence base, relative advantage, adaptability, trialability, complexity, design, and cost. For example, Desimone et al. (2002) found that training focused on specific instructional practices (e.g., an intervention) increased teachers’ use of those practices in the classroom. In particular, they reported that characteristics of the structure of the activity (i.e., reform type, duration, and collective participation) and characteristics of the substance of the activity (i.e., active learning and coherence) impact the effect of training on the teacher’s instruction. Further, in a study of the feasibility of a teacher-implemented intervention, Solari et al. (2018) concluded that intervention developers “must ensure that the interventions are streamlined and efficient as well as effective to increase the likelihood of consistent implementation” (p. 187). In other words, intervention-level determinants, such as the intervention design and complexity, play a key role in the feasibility of implementation.

Teacher-level characteristics that may serve as determinants of implementation include need (i.e., well-being or personal fulfillment that will be addressed by implementing), capability (i.e., competence, knowledge, and skills to implement), opportunity (e.g., availability, scope, and power to implement), and motivation (e.g., commitment to implementing). For example, there is evidence that teachers’ reading content knowledge impacts their instructional practices (Piasta et al., 2020; Spear-Swerling & Zibulsky, 2014) and that teachers’ self-efficacy is associated with their reading instruction (Guo et al., 2012; Varghese et al., 2016).

School-level determinants that may relate to the implementation of interventions include general characteristics of the school, such as structural characteristics, relational connections, communications, and culture. There are also school-level factors that are specific to the implementation of the intervention, including tension for change, compatibility, relative priority, incentive systems, mission alignment, available resources, and access to knowledge and information. For example, Mihai et al. (2017) conducted a qualitative analysis to understand the factors that influenced Head Start teachers’ implementation of a new literacy curriculum. They discovered that the teaching context influenced teachers’ implementation of the curriculum, with teachers indicating that competing responsibilities and other expectations impeded their ability to consistently implement the literacy curriculum (i.e., the relative priority of implementing the new curriculum was low). Notably, a key purpose of the CFIR is to retrospectively explain implementation outcomes by assessing determinants within particular contexts, which then allows for the development and implementation of strategies that may best address specific contextual determinants (Damschroder et al., 2022).

## SPELL-Links to Reading and Writing

SPELL-Links to Reading and Writing is an explicit, systematic word study intervention program. According to the publisher, the three main characteristics of SPELL-Links instruction are that it is (a) speech-to-print, (b) multi-linguistic, and (c) meta-linguistic.

SPELL-Links uses a *speech-to-print* approach that distinguishes it from more traditional print-to-speech approaches that begin with the written letter and teach students to match the letter to a sound. As a speech-to-print intervention, SPELL-Links organizes instruction around the ~ 44 phonemes of English rather than around the many hundreds of graphemes in English orthography. Students learn multiple spellings for each sound at the same time, rather than waiting to master one grapheme-phoneme correspondence before moving to the next. In other words, each activity in a SPELL-Links lesson is centered around a target sound or sounds and the spelling patterns associated with that sound or sounds. For example, in the lesson in which students learn about the short vowel /ă/ sound, they are taught that there are four ways to spell the sound: *a* (as in “mat”), *a_e* (as in “giraffe”), *au* (as in “laugh”), and *ai* (as in “plaid”). The six activities in that lesson focus on the short vowel /ă/ sound and those four spelling patterns.

SPELL-Links is *multi-linguistic* in that it integrates instruction in phonological awareness, orthography, semantics, and morphology into lessons through various word study activities, working to “simultaneously develop, connect, and integrate the different processes and regions of the brain involved in effective reading and writing” (Learning by Design, 2023). For example, the lesson that focuses on the consonant /w/ sound, with an emphasis on the consonant digraph “wh,” includes four activities. One of these activities addresses phonological awareness (i.e., segmenting sounds in words that include the phoneme /w/), two address orthography (i.e., knowledge of letter-sound relationships for the consonant /w/ sound and mental images of words that include the “wh” digraph), and one addresses semantics (i.e., knowledge of word meanings for words that, again, include this digraph).

SPELL-Links is *meta-linguistic* in that it incorporates multiple word study strategies that support independent problem-solving when spelling and reading words. Each SPELL-Links activity emphasizes the use of one or more strategies. For example, in an orthography activity in the lesson in which students learn about graphemes that spell the consonant /z/ sound, students are taught to use the “No Fouls” strategy (i.e., “I must use an allowable spelling”) to correctly spell words with /z/. In another orthography activity in that same lesson, students are taught to use the “Fix the Funny Stuff” strategy (i.e., “If my spelling of a word looks ‘funny,’ I can try different allowable spellings for a part of the word that doesn’t look ‘right’ and choose the spelling of the word that looks ‘right’”). Importantly, the intervention emphasizes the importance of metacognition and self-regulation, providing students with opportunities to reflect on their strategy use and self-evaluate their performance at the end of each activity.

### Previous research on SPELL-Links to Reading and Writing

The SPELL-Links to Reading and Writing intervention has been shown to improve encoding, decoding, and curriculum-based writing (Apel et al., 2004; Kelman & Apel, 2004; Wanzek et al., 2017). For example, Apel et al. (2004) conducted a study in which one third-grade class received the traditional school spelling curriculum and one third/fourth-grade class received instructional methods featured in SPELL-Links to Reading and Writing. Students receiving SPELL-Links instruction participated in 17 sessions focused on phonological awareness (6 sessions), orthographic principles (6 sessions), and morphological awareness (5 sessions). Results revealed that students who received the SPELL-Links instruction demonstrated significant growth in their spelling abilities, whereas the students that received the traditional curriculum did not demonstrate measurable gains in spelling. Kelman and Apel (2004) conducted a case study of a fifth-grade student with spelling difficulties. After receiving 11 sessions of instruction in orthographic knowledge and phonemic awareness using instructional methods featured in SPELL-Links, the student demonstrated significant improvement in her spelling performance. The instruction also led to an increase in the student’s word-level reading ability, even though no direct reading instruction was provided.

Wanzek et al. (2017) employed a randomized controlled trial to evaluate the effects of transcription instruction. In their study, first-grade students were randomly assigned to one of four conditions: (a) spelling instruction, (b) handwriting instruction, (c) combination spelling and handwriting instruction, or (d) no intervention. The spelling instruction group received 24 SPELL-Links lessons in small groups of four students. The SPELL-Links instruction significantly improved students’ spelling outcomes and moderate effect sizes were reported for curriculum-based writing measures. Notably, previous studies of SPELL-Links have not measured implementation fidelity (Apel et al., 2004; Kelman & Apel, 2004) or have only measured fidelity when the intervention was implemented by interventionists hired and trained by the research team (Wanzek et al., 2017).

## Study purpose

There is value in exploring the feasibility of implementing interventions in authentic classroom contexts. Therefore, we aimed to observe SPELL-Links to Reading and Writing instruction provided by reading intervention teachers of students in Grades 2 and 3 to evaluate the extent to which teachers are able to implement the intervention with fidelity. We also sought to examine the teacher-reported determinants of intervention implementation and their perceptions of the importance, feasibility, and effectiveness of the intervention. We asked:

1. To what extent are reading intervention teachers able to implement SPELL-Links to Reading and Writing with fidelity? What qualitative differences are present in the varying levels of implementation fidelity?
2. What do teachers perceive as the determinants of the implementation of SPELL-Links?
3. To what extent do teachers consider SPELL-Links to be important, feasible, and effective?

## Method

### Study context and participants

The study was approved by the research team’s institutional review board and the school district’s research department. The study was conducted during the 2023–2024 academic year. Six reading intervention teachers from four schools in one school district in Virginia participated in the study. Students in this district are primarily White (60%) and African American (25%); 64% of students in this district are economically disadvantaged (i.e., qualify for free or reduced-price lunch). Teachers were eligible to participate in the study if they provided reading intervention to small groups of elementary students. All participating teachers identified as White and female. They had an average of 10.83 years of experience as reading interventionists (range, 1–21 years). Teacher demographic information is provided in Table 1. The participating teachers were asked to choose one small group of 3–5 students in Grades 2 or 3 to receive the SPELL-Links to Reading and Writing intervention. Demographic information for the students receiving the intervention was not collected.

**Table 1:** Teacher information

	Gender	Race	Highest Level of Education	Years as a Reading Interventionist	TSELI Survey Rating	TULIP Survey Score
T1	F	W	M	20	7.55	95%
T2	F	W	M	21	6.64	97%
T3	F	W	M	4	7.27	80%
T4	F	W	M	1	7.00	100%
T5	F	W	M	1	7.82	90%
T6	F	W	M	18	6.82	95%

*F* female, *W* White, *M* master’s degree, *TSELI* Teachers’ Sense of Efficacy for Literacy Instruction (Tschannen-Moran & Johnson, 2011), *TULIP* Teacher Understanding of Evidence-Based Literacy Instructional Practices (Hall et al., 2023)

The SPELL-Links to Reading and Writing publisher, Learning by Design, provided the materials needed for the participating teachers to implement the intervention with small groups of up to six students for 30 min a day, 4 days a week, for the duration of the school year. Note that although the SPELL-Links to Reading and Writing intervention is designed for one-on-one implementation, the research team was assured by Learning by Design that it could be implemented with small groups of students.

#### Intervention training

Learning by Design provided a six-hour virtual training session prior to teachers delivering the intervention. The training included (a) an overview of the SPELL-Links materials, (b) an introduction to structured word study and the core principals of SPELL-Links (i.e., speech-to-print, multi-linguistic, and meta-linguistic), (c) information on the speech-to-print approach and why teaching spelling is important to overall writing and reading skills, (d) directions on how to prepare for and implement the SPELL-Links activities in small groups, (e) directions on how to administer, score, and interpret SPELL-Links assessments, and (f) information about additional SPELL-Links resources. Once teachers began implementation, they were offered one additional, optional 30-min meeting with the training provider to ask questions about implementation. Additionally, teachers were provided with access to an online professional learning community and were encouraged to utilize the online platform as a resource for implementation suggestions.

### Data collection and analysis plan

#### Implementation logs

Participating teachers completed logs in which they documented their implementation *dosage* (i.e., they recorded the SPELL-Links activity taught each day and the amount of time spent implementing SPELL-Links each day). Teachers documented reasons they did not implement the intervention on some days (e.g., no school, field trip, teacher absence, testing). These logs allowed us to determine whether teachers implemented the intervention according to the recommended schedule of four days per week. Teacher implementation log data was analyzed descriptively.

#### Implementation observations

To measure *adherence* and *quality,* we videorecorded teacher implementation on three separate occasions during the approximately 20-week study period. We employed a researcher-developed adherence and quality measure aligned to the SPELL-Links intervention. Each SPELL-Links activity provides the teacher with step-by-step directions. The *adherence* section of the measure outlines these activity steps. Each activity step was coded as 1 (implemented) or 0 (not implemented). The *quality* section of the measure included four quality of implementation indicators (i.e., accuracy of modeling, scaffolding, pacing, and preparation/organization) for each activity. Each quality indicator was coded on a 3-point Likert scale in which 3 = high quality, 2 = moderate quality, and 1 = low quality. The measure also included a section for a written summary or important notes about the activity. Supplemental Appendix A provides further details regarding the definitions, expectations, and coding guidance for each indicator.

Each observation was coded for adherence and quality using the researcher-developed measure. To analyze adherence and quality, we calculated the number of points that would be awarded for perfect implementation and the percentage of this perfect score obtained by each teacher. For example, in an activity in which the teacher implemented all activity steps except for Step 2, she received an adherence score of 11 out of 12, or 92% adherence. These scores were averaged across observations and across teachers to determine the extent to which teachers are able to implement SPELL-Links with adherence and quality. In alignment with Hill and Erickson (2019), we considered scores of less than 50% as “low,” scores of more than 80% as “high,” and scores in between (i.e., 50–80%) as “medium” (p. 592).

Prior to coding, coders participated in a one-hour training delivered by the first author, a researcher with extensive coding experience. The training was followed by a practice session during which coders watched a video, coded the session independently, and then discussed codes. Inter-observer agreement was established prior to coding video-recorded observations, with the first author serving as the “gold standard” and establishing a set of correct observation codes against which other observers’ codes were compared. Percent agreement was calculated as the number of agreements divided by the total number of possible codes. Coders were required to reach 90% agreement prior to coding observations independently (*M* = 94%). Approximately 50% of observations were double-coded. The average agreement across double-coded observations was 94%. Discrepancies in coding were resolved via discussion and consensus.

#### Interviews

At the end of the intervention period, teachers participated in semi-structured videoconference interviews (see Supplemental Appendix B for the interview guide). Semi-structured interviews are commonly used as a flexible approach to gather in-depth information about participants’ perceptions (Creswell & Poth, 2017). The use of semi-structured interviews allowed us to engage in conversations with teachers related to the determinants of implementing the intervention. They also enabled us to ask follow-up questions or provide probes that encouraged teachers to elaborate on their responses.

To identify determinants of implementation, the teacher interviews were audio recorded, transcribed, and analyzed thematically. Thematic analysis is commonly used to analyze the experiences and perspectives of research participants (Braun & Clarke, 2006). We used a priori domains from the CFIR framework (Damschroder et al., 2022) to deductively code the teacher interviews. As previously mentioned, the CFIR framework is a comprehensive list of factors related to implementation (e.g., beliefs about capabilities, environmental context and resources). Two coders independently analyzed each interview and discrepancies were resolved via discussion and consensus. By comparing teachers’ responses to the questions, we could identify patterns, or themes, in the data and explore differences in teachers’ responses relating to intervention feasibility. Table 2 provides examples of how CFIR constructs were used to analyze teacher interview responses and identify themes.

**Table 2:** Examples of using the CFIR to analyze interview data

CFIR construct	CFIR definition	Interview example	Assigned theme
Access to knowledge & information	Guidance and/or training is accessible to implement and deliver the innovation	“There is a lot packed into that one day, and it seems like there were some things that we could have spent more time on” (T6)	Barrier: intervention training
Compatibility	The innovation fits with workflows, systems, and processes	“It was just too much time and energy for me to devote for one 30-min group when I have nine groups” (T5)	Barrier: compatibility with existing practices
Mission alignment	Implementing and delivering the innovation is in line with the overarching commitment, purpose, or goals in the inner setting (school/classroom)	“I was never going to get to where I needed to get for my second graders to be able to pass that PALS spelling” (T3)	Barrier: alignment with goals
Capability	The individual(s) has interpersonal competence, knowledge, and skills to fulfill role (innovation deliverer)	“I already had a lot of background about the skills I was teaching, so I think that helped” (T4)	Facilitator: teacher capability

*CFIR* Consolidated Framework for Implementation Research (Damschroder et al., 2022)

#### Surveys

Prior to receiving training and intervention materials, teachers completed the Teacher Understanding of Evidence-Based Literacy Instructional Practices (TULIP) survey (Hall et al., 2023) and the Teachers’ Sense of Efficacy for Literacy Instruction (TSELI) survey (Tschannen-Moran & Johnson, 2011). The TULIP survey assesses teacher knowledge in the domains of (a) phonological awareness, (b) phonics, decoding, and encoding, (c) reading fluency, (d) oral language, and (e) reading comprehension. In their validation study, Hall et al. (2023) reported a Cronbach’s alpha reliability estimate of 0.93. The TSELI survey asks teachers to respond to 22 items on a 9-point Likert scale (1 = not at all; 9 = a great deal) by considering the combination of their current ability, resources, and opportunity in their present position. The TSELI survey has a Cronbach’s alpha reliability estimate of 0.96 (Tschannen-Moran & Johnson, 2011). At the end of the study period, teachers completed a survey to assess their perceptions of the importance, feasibility, and effectiveness of the intervention. Teachers were presented with 16 items and asked to rate each item on a 5-point Likert scale (1 = strongly disagree; 5 = strongly agree). Teachers were also prompted to share any additional feedback through an open-ended item at the end of the survey. The data collected via these surveys allowed us to better describe our study sample and triangulate our findings. All survey data was analyzed descriptively.

## Results

### Recruitment and retention

During the initial recruitment phase for this study in May 2023, 10 reading intervention teachers consented to participate. Before the study began, two teachers withdrew because they were moving to new school districts and one additional teacher consented to participate. Nine teachers participated in the virtual SPELL-Links training in September 2023. After receiving the training but before beginning to implement the intervention, three teachers withdrew from the study. Teacher reasons for withdrawal included feeling like implementing SPELL-Links was “too much to handle” and not feeling like they can “teach this program with the energy and focus that it deserves.” One teacher, who was new to the school and position, noted that it was “not the best time for [her] to try a new program.” Six teachers began implementing the intervention in October 2023. In January 2023, one teacher (T3) decided to discontinue implementation of SPELL-Links with her students because she perceived that her students had “not grown since beginning this program.” However, she agreed to participate in an interview and complete a survey so that we could learn about her experiences with the intervention.

### Fidelity of implementation

Individual teacher implementation fidelity data is reported in Table 3.

**Table 3:** Implementation fidelity

	Dosage*	Average adherence	Average quality
T1	50 (2.5)	81%	97%
T2	48 (2.4)	88%	89%
T3	23 (2.1)	73%	92%
T4	53 (2.7)	100%	100%
T5	44 (2.2)	85%	100%
T6	65 (3.3)	80%	89%
Average	47 (2.5)	85%	95%

^*^Dosage = total number of sessions implemented (average number of sessions implemented per week)

#### Dosage

In terms of *dosage*, teachers were expected to implement SPELL-Links activities 4 days a week for 30 min per day as prescribed by Learning by Design. For the teachers who implemented the intervention for all 20 weeks of the study period, the average implementation dosage was 52 sessions (range, 44–65). Thus, they implemented 2.60 sessions per week on average. The teacher who only used the intervention for 11 weeks implemented 23 sessions, for an average of 2.09 sessions per week. In their implementation logs, teachers recorded when they did not implement the intervention and their reasons for not implementing it. The most reported reasons for not delivering the intervention were no school/early release (average of 24 instances across teachers), teacher was unavailable (e.g., due to training, teacher absence; average of 10 instances), and student testing (average of 8 instances). Regarding student testing, it is worth noting that several teachers administered intervention-aligned assessments frequently (i.e., approximately every 2–3 weeks) to assess their students’ mastery of the intervention content. Additionally, teachers reported that although they pulled their groups for 30 min, only about 25 min were spent on instruction. Based on the observation videos, the average session length was 24.55 min (range, 15.75–29.75). Overall, teachers were not able to implement the intervention at the intended dosage of 30 min per day, 4 days per week.

#### Adherence and quality

Data from observations demonstrated that teachers adhered to 85% of activity steps on average (range, 67–100%). Across teachers, average adherence ratings ranged from 73 to 100%, indicating moderate to high levels of adherence (Hill & Erickson, 2019). The average quality of delivery rating across the observations was 95% (range, 83–100%). Across teachers, average quality ratings ranged from 89 to 100%. Thus, average quality ratings exceeded 80% for all teachers, indicating high instructional quality (Hill & Erickson, 2019).

#### Qualitative differences

Based on our qualitative data (i.e., written summaries) from observation coding, we are able to construct a better understanding of teachers’ fidelity scores. For example, one teacher (T5) whose average adherence across the three observations was 85% had individual observation adherence scores of 100%, 70%, and 86%. This large variance in adherence scores is unusual (especially in combination with an average quality score of 100%) and warranted further exploration. The qualitative data for this teacher’s observations revealed that she frequently adapted activities based on the needs of her students. For example, in one activity, the coder noted, “The teacher skipped Steps 5–7, but it seemed intentional. Her students did not seem to need the intermediate steps of having the first letter present before spelling the whole word, and completing all the steps would have taken too much time. Therefore, this felt like a successful adaptation to the activity.” However, making this adaptation involved skipping activity steps and ultimately resulted in a lower adherence score for this activity.

We also explored how teachers with varying levels of adherence and quality implemented similar activities. For example, two teachers (T4 and T6) implemented the same activity (12.1 “Lines & Letters”) during their second observation. T4 implemented the activity with 100% adherence and quality. The qualitative summary of her implementation of this activity stated, “The teacher really seemed to understand the activity and provided additional information to support her students’ learning. Before starting the activity, she did a review of letter sounds. During the activity, she modeled before students practiced (i.e., used explicit instruction). She also gave students additional ‘challenge’ words to practice (i.e., upward scaffolding).” On the other hand, T6 implemented the activity with 75% adherence and 83% quality. Her qualitative summary stated, “It felt like she rushed into the activity and did not provide much explanation about the objective of the activity as directed in Step 1. She delivered Steps 2–5 well but skipped Step 6 and only discussed one of the two strategies for Step 8. Together, these actions indicate that she was not well prepared to implement the activity.” These stark differences in activity adherence and quality make it worthwhile to explore the determinants of implementation.

### Determinants of implementation

The primary *barriers* to implementation that we identified from teacher interviews and surveys were related to intervention training, intervention content and structure, compatibility with existing practices, and alignment with goals. The primary *facilitators* we identified were teacher capability (e.g., knowledge, self-efficacy) and support from other teachers. We detail each of these determinants below, noting how these factors related to implementation fidelity.

#### Barrier: intervention training

All six teachers indicated in their interviews that they perceived the intervention training as insufficient and that more training would have helped them implement the intervention more successfully. When asked about the training, one teacher with a low dosage, high adherence, and high quality shared, “I felt like it was not enough, and also crammed for one day, like it was really long, and I think it would have been better to have an in-person training versus a Zoom training, and that it could have been broken up over at least two days, maybe more” (T5). A teacher with slightly lower adherence but high quality similarly mentioned, “It was a lot in one day … if it was in smaller chunks, it probably would have been better so I could have tried something and then gone back for further training on it” (T1). Another teacher with moderate adherence further explained, “there is a lot packed into that one day, and it seems like there were some things that we could have spent more time on, and kind of spread out some of that” and “if we had time to actually do some of the activities together, and actually take more time to kind of investigate and figure all that out, because a lot of it we had to figure out on our own” (T6). Even a teacher with very high adherence and quality shared similar sentiments. She noted that the training “probably should have been longer” and that “it would have been good to sleep on it and come back” (T4).

Data extracted from the online professional learning platform indicated that participating teachers visited the site an average of 22 times (range, 2–63 visits). When asked during interviews about their use of the online community, most teachers indicated that they tried using it, but it was “overwhelming” (T6; 63 visits) and “complicated” (T2; 25 visits). One teacher said, “I had a hard time navigating it. I tried.” (T3; 18 visits). Ultimately, it appeared that teachers felt it was “too much to deal with” (T5; 4 visits) and no teacher described successfully using it as a resource to support their implementation. Overall, teachers did not perceive the training they received as sufficient in supporting their implementation of the intervention.

#### Barrier: intervention content and structure

All six teachers indicated in their interviews that the intervention content and structure were not ideal. Notably, they perceived the intervention as complex, rather than straightforward and efficient, to implement. A teacher with high adherence and very high quality of implementation explained, “It was an overwhelming amount of information, and even if you read ahead, prepped, and did what you were supposed to, and had everything out and read ahead of time, still you didn’t feel like you knew what you were really doing” (T5). Another teacher with similar levels of adherence and quality elaborated, “Even though I would read through the lessons multiple times before I did it, I found it wasn’t easy to move through. It wasn’t always natural. … I just always was not positive I was doing the right thing” (T1).

Additionally, the intervention was designed for one-on-one implementation, but all teachers in our sample used the intervention with a small group of students and thus were required to adapt the intervention for small-group instruction. One teacher with high adherence and high quality explained, “It made it a little more complicated when you read the directions to be like, ‘Okay, I’m not doing this with one kid, so now I just need to divvy things up equally, or I need to, like I said, we’re not back and forth, back and forth, we’re going around the table. It wasn’t super hard to do, but it’s a harder lesson. It’s not that hard to change it like mentally in your head, but it was harder to just implement in general than if it had been one on one” (T5). Another teacher shared, “I would do kind of a mix between what it said for small group and what it said [for one-on-one]. My students don’t always work well together because of their personalities. … So, instead of putting words on cards, I would put it on the board, and then we talk about it on the board together, and we fix it together, and then we’d use their notebook. … but we would do a lot using the board instead of individual cards” (T1). Notably, this teacher had relatively moderate adherence despite having high quality of delivery. Based on the qualitative data from the observations, this teacher’s adaptations to small group instruction occasionally led to low adherence to the activity steps.

In sum, teachers in our study perceived the intervention as overwhelming and complex to implement, even when they prepared ahead of time. One particular area of difficulty was the need for teachers to adapt the one-on-one intervention for small-group instruction. Taken together, the intervention content and structure posed a challenge to teachers’ implementation.

#### Barrier: compatibility with existing practices

Some teachers noted the lack of compatibility with their existing practices (e.g., planning time) as a perceived barrier to implementation. For example, a teacher with moderate dosage who implemented the intervention with very high adherence and quality said, “It was so different from what we’re already doing that it just felt like it was unknown. … It took a lot of time to prep. … It was just tough for this year, and the structure of our intervention, I didn’t feel like it really fit with what we were already doing. And I think that’s the part that I had the hardest time with” (T4). Another teacher with low dosage, high adherence, and very high quality said, “It was just too much time and energy for me to devote for one 30-min group when I have nine groups” (T5). She further explained, “It was helpful that our planning was right before this group… I would use part of that time every day to prep, which is more than I had to do for any of my other groups because [for those groups] I could do my lesson plans for the week, know what to print, know what to read, and everything is pretty much ready, whereas this I felt like it was too much to prep every single day.” Notably, even the teacher with the highest dosage said, “if you’re really going to be committed to it… you need more time to have the commitment” (T6). As such, the extensive time needed to prepare for implementing the intervention was not compatible with the existing practices of our teachers.

#### Barrier: alignment with goals

A few teachers also expressed a lack of alignment between the intervention and their goals for student performance. In particular, the pacing of the intervention was a concern. One teacher with low dosage shared, “I felt like it was going really slow. And I was never going to get to where I needed to get for my second graders to be able to pass that [state screening measure of] spelling” (T3). Another teacher with low dosage further explained, “It felt like it was really slow to get through what we’re getting through. I’m still on digraphs and it’s February. I've gotten through short vowels, and I’m not even through digraphs, and that’s all we’ve done, when in other programs I could have knocked out that in November and been on to the next thing” (T5). A teacher with moderate dosage but very high adherence and quality similarly noted, “I would have liked for it to move a little bit faster” (T4).

Teachers also noted that the intervention did not focus on the skills they thought were most relevant for their students. For example, one teacher mentioned, “I would like it if it had more reading… [there] is not a whole lot of [reading] in context” (T1). Another teacher shared, “I didn’t feel like there was enough reading involved. It was like a lot of spelling and some reading, but it wasn’t reading in context at all” (T5). The same teacher also said, “I feel like it’s too much of a focus on just one thing, instead of getting through multiple things in a day, being able to do warm up things, being able to read, being able to write and spell.” One teacher with very high adherence and quality but moderate dosage mentioned, “I did supplement some materials because I didn’t feel like it was always enough for my intervention babies” (T4).

Teachers also expressed that they did not perceive the instructional practices used in the intervention as efficiently moving their students toward their goals. One teacher said, “I think my intervention babies need more explicit [instruction]” (T4). She further described, “It felt a little bit like they were doing a lot of discovery learning … but I think with the intervention kids, they need all the support and scaffold that they can get, and just kind of asking them to discover it, I don’t necessarily think is the best way to get there in intervention” (T4). Overall, the alignment between the intervention, especially the pacing, the skills addressed, and the instructional practices used, and their students’ performance goals was an area of concern for teachers.

#### Facilitator: teacher capability

We identified teacher capability (e.g., knowledge, skills, self-efficacy) as a facilitator of implementation. For our sample of teachers, the average percent correct on the teacher knowledge survey was 93% (range, 80–100%), indicating that teachers demonstrated a high level of knowledge of English sound structure, orthography, and evidence-based literacy instructional practices. There is a noteworthy pattern in the fidelity data related to knowledge. The two teachers with the highest knowledge survey scores (T4, 100%; T2, 97%) received the highest average adherence scores (T4, 100%; T2, 88%) whereas the teacher with the lowest knowledge survey score (T3, 80%) received the lowest average adherence score (73%). This finding suggests that teachers with higher levels of knowledge may be able to better adhere to the activity steps for this intervention. Additionally, the average self-efficacy survey rating was 7.18 out of 9.00 (range, 6.64–7.82), indicating moderate to high levels of self-efficacy for providing literacy instruction. There is also a noteworthy pattern in the fidelity data related to self-efficacy. The two teachers with the lowest self-efficacy ratings (T2, 6.64; T6, 6.82) also received the lowest average quality of implementation scores (both 89%), suggesting that lower self-efficacy may lead to lower quality of implementation.

Our interviews revealed similar findings. For example, the teacher with the highest adherence (T4) noted she felt “pretty confident” implementing the intervention. She explained, “doing reading intervention, we already know what they need, and we’re used to breaking it down by skill, and I already had a lot of background about the skills I was teaching, so I think that helped.” Conversely, some of the lower adherence teachers said they were “only mildly” (T1) or “not real” confident (T6), or that their confidence “depended on the activity” (T3). Taken together, these findings suggest that teacher knowledge and self-efficacy impacted their implementation of the intervention.

#### Facilitator: peer support

Three of our teachers were located at the same school. They described being able to discuss the intervention during lunch and planning periods together as “beneficial” to their implementation (T5). One teacher explained, “we got on a flow for a while where we would be within a couple of days of each other, so it would be like, ‘okay, I made these cards, let me pass them to you’ or you’d be like, ‘okay, I looked at this, and I don’t understand it, did you?’” (T4). Another teacher shared, “doing it together has helped with getting through it” and “it really does work better if you have that right there in your school” (T6). Overall, these three teachers implemented the intervention with moderate to high dosage. The average adherence scores for these teachers ranged from 80 to 100% and their average quality scores ranged from 89 to 100%. As such, peer support facilitated high-fidelity intervention implementation.

### Teacher perceptions of the intervention

Based on the teacher survey of the importance, feasibility, and effectiveness of the intervention, teachers had varying perceptions of the intervention. We present means and range scores below; however, the individual teacher ratings in Table 4 offer a more comprehensive view of these data.

**Table 4:** Teacher survey ratings

	T1	T2	T3	T4	T5	T6	Average
The literacy skills targeted in the program are important	4	5	5	4	3	5	4.33
The activities (e.g., Tap and Map, Sort It Out, Picture This) were appropriate for my students	4	4	1	4	3	5	3.50
I was satisfied with the program	3	3	1	2	4	3	2.67
I was confident in my ability to implement the activities	3	3	2	4	5	3	3.33
The activities were easy to implement	4	4	3	2	5	2	3.33
The activity materials were easy to use	3	3	3	2	3	2	2.67
I could complete the activities in a reasonable amount of time	4	4	3	2	4	3	3.33
I could implement the activities without extensive preparation	3	4	4	2	4	2	3.17
The training and online platform (SPELL-Link’d) provided me with adequate support to implement the program	2	5	1	2	4	2	2.67
The program improved my students’ abilities to read words	3	3	4	4	3	3	3.33
The program improved my students’ abilities to write words	4	3	1	2	3	3	2.67
I think continuing to use the activities with my students will make them better readers	4	3	2	2	5	3	3.17
I think continuing to use the activities with my students will make them better writers	4	4	1	2	5	3	3.17
I believe the learning that occurred during this program will help my students be successful in the upcoming years of education	4	3	1	3	4	3	3.00
I have noticed that my students have generalized the skills they learned from the program to other environments and contexts	3	3	2	3	5	2	3.00
I think this program holds promise for other students	4	2	1	2	4	3	2.67
Average	3.37	3.47	2.37	2.63	4.05	2.95	3.14

1 = strongly disagree; 5 = strongly agree

#### Perceptions of importance and feasibility

On a 5-point scale (1 = strongly disagree; 5 = strongly agree), teachers indicated that they agreed that the literacy skills targeted in the intervention are important (*M* = 4.33; range, 3–5). They also somewhat agreed that the activities in the intervention were appropriate for their students (*M* = 3.50; range, 1–5). However, their overall satisfaction with the intervention was rather neutral (*M* = 2.67; range, 1–4). Notably, teachers indicated that they did not feel like the training and online platform provided them with adequate support to implement the intervention (*M* = 2.67; range, 1–5). Accordingly, teachers were only somewhat confident in their abilities to implement the activities (*M* = 3.33; range, 2–5). Regarding feasibility, teachers slightly agreed that the activities were easy to implement (*M* = 3.33; range, 2–5), could be completed in a reasonable amount of time (*M* = 3.33; range, 2–4), and could be implemented without extensive preparation (*M* = 3.17; range, 2–4), but they did not agree that the activity materials were easy to use (*M* = 2.67; range, 2–3). In the open-ended response section of the survey, one teacher with high dosage but moderate adherence explained, “If the training was face to face and more explicit, I feel I would have felt better about implementing the program” (T6).

#### Perceptions of effectiveness

Teachers somewhat agreed that the intervention improved their students’ abilities to read words (*M* = 3.33; range, 3–4) but did not agree that it improved their students’ abilities to write words (*M* = 2.67; range, 1–4). They also did not agree that the intervention holds promise for other students (*M* = 2.67; range, 1–4). Several teachers provided more information about their perceptions of the intervention in the open-ended response section of the survey. For example, one teacher shared, “I have a strong literacy background, so there are many phonics tools in my literacy toolbox. This program had some positive components, but I believe that there are more activities and strategies needed in our reading intervention setting” (T2). One teacher mentioned, “The program was not systematic and explicit enough for my students, and too many ways to read or spell words were given at a time” (T3). Another teacher similarly noted, “I think that at times this program gives too much information at once” and “this program was not explicit enough in the spelling” (T1).

## Discussion

The purpose of this study was to explore the extent to which reading intervention teachers who were provided a one-day training without ongoing coaching support were able to implement the SPELL-Links to Reading and Writing intervention with fidelity. It also aimed to better understand the determinants of teachers’ implementation of the intervention and their perceptions of the importance, feasibility, and effectiveness of the intervention.

### Exploring determinants of implementation fidelity

On average, teachers implemented the SPELL-Links intervention with high adherence to activity steps and delivered it with high quality (i.e., scores greater than 80%; Hill & Erickson, 2019). However, as a group, they did not implement it at the intended dosage of 4 days per week. This finding is not unexpected given that intervention research has consistently demonstrated difficulties with achieving the intended dosage (Denton et al., 2021; Solari et al., 2018). Yet it is problematic because some evidence suggests that implementing interventions with intended dosage is associated with improved outcomes (Vadasy et al., 2015; Wolgemuth et al., 2014). Therefore, it is worthwhile to explore the determinants of implementation to better understand how we can support teachers’ successful intervention implementation.

#### Intervention-level determinants

At the intervention level, our findings suggest that the training was identified as a barrier to implementation. Although the use of a one-day standalone training with limited follow-up is common, research has shown that there are limitations to one-day training (e.g., Desimone et al., 2002; Garet et al., 2001). Without structured, ongoing implementation support (e.g., coaching) teachers may show low levels of fidelity (Guskey & Yoon, 2009). Thus, the fact that participating teachers were only provided with a one-day training without ongoing coaching may be one reason for lower levels of fidelity. In a meta-analysis on the effects of teacher training on intervention implementation, Brock and Carter (2017) estimated that training has a strong effect on implementation fidelity (*g* = 1.08). That said, research suggests that successful teacher training builds upon background knowledge, explicitly describes and illustrates content, provides opportunities to actively apply and generalize learned content in real-world contexts, and supports metacognition (e.g., reflection and self-monitoring) throughout the training process (Trivette et al., 2009).

Our findings also suggest that the content and structure of the intervention were a key barrier to implementation. This barrier was not surprising given that previous research has highlighted the importance of developing interventions that are streamlined and efficient (Solari et al., 2018). Further, in a study on teachers’ perceived barriers to intervention implementation, Long et al. (2016) revealed that over half (58%) of the teacher-identified barriers to implementation were perceived to be related to the intervention itself. Teachers in our sample perceived the SPELL-Links intervention to be complex and overwhelming. They shared that even when they prepared ahead of time, they still felt like they did not know what they were doing. It is important to note that these teachers had previously been implementing interventions designed according to print-to-speech principles. Print-to-speech approaches to instruction organize instruction around graphemes, introducing each grapheme (usually starting with simple, single-letter graphemes) and describing the phoneme that corresponds to it. As mentioned previously, the SPELL-Links intervention utilizes a less traditional approach to instruction. The speech-to-print approach used in SPELL-Links organizes instruction around the ~ 44 phonemes of spoken English. Teachers introduce a given phoneme and describe all of the different graphemes that correspond to that phoneme. As such, the implementation of SPELL-Links required a paradigm shift for participating teachers. Although we cannot determine how much of their perceptions of implementing the intervention were related to this paradigm shift, it may at least partially explain teachers’ feelings that the intervention was difficult to implement.

#### School-level determinants

Three school-level determinants were identified in our data: compatibility with existing practices, alignment with goals, and peer support. Notably, two of these determinants (compatibility with existing practices and alignment with goals) served as barriers, whereas peer support served as a facilitator. It is also worth noting that school-level factors are usually not within the teacher’s control. They often fall within the responsibilities of school administrators.

The lack of compatibility with existing practices was primarily evident through the perceived large amount of planning and preparation required for implementing the intervention. This finding is in alignment with previous research on barriers to implementation, which has demonstrated adequate planning time is a prerequisite for high-level implementation (Long et al., 2016) and that competing priorities can make it challenging to find time to implement (Mihai et al., 2017). In other words, when teachers do not have enough time to plan and prepare, their implementation of the intervention suffers.

The lack of alignment with the teachers’ goals for their students was another significant school-level barrier to intervention implementation. Teachers often cited concerns regarding the inability of the intervention to adequately address the skills their students needed to master to meet state-specified benchmarks within the allotted timeframe. Given that prior research has shown that teachers are more motivated to implement an intervention when they believe it will improve student outcomes (Cramer et al., 2023), it follows that the teachers in our sample did not implement the intervention at the intended dosage. It is important to note, though, that we did not measure student outcomes in the current study. It is also worthy to note that the teachers in our sample, on average, were experienced reading interventionists, demonstrated high levels of knowledge to teach literacy, and reported moderate to high levels of self-efficacy for teaching literacy. Therefore, we posit that they were often able to use their experience, knowledge, and skills to overcome this barrier. For example, one teacher (T4), who described the intervention as not being explicit enough and not having enough practice and review for her students, explained that she was able to adapt the intervention and “supplement” with materials she thought were more appropriate for her students, while still providing the intervention as intended (i.e., average adherence and quality was 100% for this teacher).

Our findings suggest that peer support could serve as a facilitator of intervention implementation. The teachers in our study reported that having other teachers at their school who were also using the intervention was beneficial to their implementation. This finding aligns with existing research showing the importance of the collective participation of teachers from the same school (Desimone et al., 2002). Additionally, previous research suggests that having a strong support system within their schools increases teachers’ sense of efficacy (Dahl-Leonard et al., 2023b) and that teacher self-efficacy is associated with improved instructional quality and student performance (Guo et al., 2012; Varghese et al., 2016). Therefore, it is possible this perceived support influenced their implementation fidelity as well as student outcomes.

#### Teacher-level determinants

Based on findings from prior research (Guo et al., 2012; Piasta et al., 2020), we anticipated that teachers’ capabilities (e.g., knowledge, skills, self-efficacy) would emerge as a determinant of implementation. We found that teacher knowledge and self-efficacy were both facilitators of high-fidelity implementation in this study. In particular, teacher knowledge appeared to align with adherence to activity steps and teacher self-efficacy appeared to be linked to quality of implementation. We also believe that our teachers used their experience, knowledge, and skills to overcome several barriers to implementation. It is worth noting that one teacher (T4) scored 100% correct on the knowledge survey and described feeling “pretty confident” in implementing the intervention due to her background. She was the only teacher to achieve an average of 100% adherence and quality. As described previously, this teacher was able to make successful adaptations to the intervention when she felt it did not provide her students with the knowledge and skills that they needed.

### Teacher perceptions of intervention importance, feasibility, and effectiveness

The results from the teacher survey on the importance, feasibility, and effectiveness of the intervention mostly aligned with our observation and interview data. For example, on the survey, teachers somewhat agreed (*M* = 3.50) that the activities in the intervention were appropriate for their students, with ratings ranging from 1 (strongly disagree) to 5 (strongly agree). The average rating for the item “I was satisfied with the program” was 2.67, with ratings ranging from 1 to 4, indicating no teachers strongly agreed with that statement. Our interviews similarly revealed that not all teachers were satisfied with the intervention content and structure. For example, one teacher acknowledged, “I know that everything I’m saying is pretty negative. I overall have a negative feeling towards the program” (T5). It was also apparent from the survey and interview data that teachers were not satisfied with the intervention training they received, and as a result, they were only somewhat confident in their ability to implement the intervention successfully. As previously mentioned, this lack of confidence has implications for implementation quality.

On average, teachers’ perceptions about the feasibility of implementing the intervention were relatively neutral. Survey results indicated that teachers slightly agreed that the activities could be completed in a reasonable amount of time (*M* = 3.33) and without extensive preparation (*M* = 3.17). Interviews with teachers demonstrated similarly neutral perceptions but did reveal that the amount of preparation required to implement the intervention was difficult for some teachers to manage. For example, one teacher shared, “It was just too much time and energy for me to devote for one 30-min group when I have nine groups” (T5). This lack of compatibility with existing school-level systems (e.g., planning time) was an identified barrier to high-fidelity implementation.

Lastly, teachers demonstrated mixed perceptions about the effectiveness of the intervention. As previously noted, one teacher (T3) decided to stop using the intervention after 11 weeks because she was not seeing student growth. In her interview, she expressed concerns that if she continued to use the intervention, then her students would not meet state-specified benchmarks, especially for spelling. Survey data similarly indicated that, on average, teachers did not see improvement in their students’ abilities to write words. However, it also indicated that teachers did see improvement in their students’ abilities to read words on average. That said, the average rating for the item “I think this program holds promise for other students” was 2.67, with ratings ranging from 1 to 4, indicating that teachers did not agree with this statement on average. Again, this is problematic because teachers are more motivated to implement an intervention when they believe it will improve student outcomes (Cramer et al., 2023).

### Limitations

There are some limitations to this study. First, the participant sample only included six teachers from one school district. All six teachers demonstrated high levels of knowledge and self-efficacy (i.e., capability) prior to engaging in the intervention training and implementation. Therefore, the reported findings are exploratory and may have limited generalizability to other populations of teachers. We also did not collect student-level demographic or outcome data. As such, we have limited information about the students that participating teachers were serving and are unable to interpret our results within the context of the student population. In our future research, we plan to collect and report demographic data as well as student performance data. This data will better enable researchers and practitioners to make determinations about the extent to which the present findings may generalize. Given a large sample of teachers, we will also be able to examine the extent to which specific barriers and facilitators may be experienced by different groups of teachers.

Additionally, a primary data collection method utilized in the present study was semi-structured interviews, which has strengths and weaknesses. Although semi-structured interviews are commonly used as a flexible approach to gathering information about participants’ perceptions, this interview method inherently lacks the rigor of structured interviews (Creswell & Poth, 2017). In the present study, we did not ask all participants the exact same questions or prompt them to elaborate on their responses in the exact same way. We also acknowledge that it would have been helpful to ask teachers more questions during the interviews. In particular, asking questions about how they used their capabilities (e.g., knowledge, skills, self-efficacy) to inform decisions around making adaptations to the intervention would have provided us with more insight on this topic. It may have also been useful to ask teachers about how the training on and implementation of the SPELL-Links intervention compared to their experiences with training on and implementation of other interventions. This information would have allowed us to better contextualize our data. Another potential limitation of interviews, especially with such a small sample of teachers, is that teachers may be more likely to report intervention-level issues rather than individual-level or school-level factors that negatively affect their implementation. This reluctance to discuss school-level factors may stem from concerns about being perceived as critical of their peers or supervisors. That said, in many instances, interview data was paired with observation and survey data to triangulate our findings.

It is also worth noting that some activities included as few as eight steps and all steps were equally weighted during the observation coding. In many instances, missing one activity step could result in a much lower adherence score (e.g., implementing 7 out of 8 steps equals 88% adherence). It is possible that a more nuanced measure, perhaps one in which activity steps are weighted differently depending on their importance, would have provided more detailed information on teachers’ adherence.

### Implications and future directions

Taken together, these results suggest there was some incongruence between teachers’ implementation and their perceptions of the intervention. Overall, teachers implemented the program with high adherence and quality. Intervention dosage was lower, but this was primarily due to factors outside of the teachers’ control (e.g., school closure). However, only one teacher agreed with the statement, “I was satisfied with the program” and no teachers agreed with the statement, “The activity materials were easy to use.” These findings suggest there may be opportunities for improvement in the development of SPELL-Links. That said, it is important to note the context for these findings. For example, teachers in the present study participated in a one-day training without ongoing support. Given all that is known about the limitations of one-day training (e.g., Desimone et al., 2002; Garet et al., 2001), these findings may not be surprising. It is possible that future studies evaluating SPELL-Links that provide ongoing, job-embedded training may yield different results. It is also worth noting that the intervention was designed for one-on-one implementation, but the teachers in our study used the intervention with small groups of students. Teachers may have implemented and/or perceived the intervention differently if it were implemented in a different context, and future studies may benefit from exploring these differences.

There are several practical implications of our findings for overcoming implementation barriers. Results suggested barriers to implementation frequently related to intervention content and structure. In particular, teachers reported that the intervention was “overwhelming” and “not easy to move through.” Therefore, intervention developers should consider ways to ensure that intervention is straightforward and efficient for teachers to implement. For example, intervention developers may consider limiting the number of different activities in the intervention and incorporating routines into the activities so that teachers feel more confident implementing the intervention. They should also ensure that the intervention training and support teachers receive fully prepare them to implement the intervention in the classrooms. Learning by Design has already taken steps to revamp the training and support they offer to teachers implementing SPELL-Links. For example, they now offer student-centered training and coaching for implementation, which features more bite-sized training sessions with opportunities for teachers to practice with a student before moving to the next training piece.

Our findings suggest that administrators adopting new interventions should select interventions that are compatible with existing practices at their school and that have sufficient alignment with school-, district-, and state-level goals for students. They should also strive to create environments in which teachers implementing the same intervention have time to plan and discuss the intervention with one another, as our findings show that teachers may benefit from having a support system within their schools. Also, it may be worthwhile for administrators to provide teachers with more opportunities to gain knowledge and skills and increase their self-efficacy, via training and ongoing support when implementing a new intervention.

Overall, this study demonstrated that reading intervention teachers were able to implement the SPELL-Links to Reading and Writing intervention with high adherence and quality, but not at the intended dosage. Examination of the determinants of implementation is crucial in supporting teachers’ implementation of such interventions. However, the present findings lack generalizability and should be considered exploratory rather than conclusive. The field would benefit from future studies that incorporate a larger, more diverse sample of participants. Further, the addition of student-level data could allow for more sophisticated data analysis, such as exploring associations between teacher implementation fidelity (e.g., dosage, adherence, and quality) and student reading achievement.

## Supplementary Information

Below is the link to the electronic supplementary material.

Supplementary file1 (DOCX 19 KB)

## Data Availability

The data that support the findings of this study may be available from the corresponding author upon reasonable request.
