# Supplementary material for: Exploring the feasibility of implementing the SPELL-Links to Reading and Writing intervention
Source: Ann Dyslexia. 2024 Aug 30;75(1):71–95. doi: 10.1007/s11881-024-00315-w (PMC11954702; doi:10.1007/s11881-024-00315-w)
Supplement: Supplementary file 1 — Supplementary file1 (DOCX 19 KB) [file 11881_2024_315_MOESM1_ESM.docx]

**Supplemental Appendix A**

Fidelity Coding Guide

**Fidelity Coding Guide**

General information:

You will code for fidelity using recorded videos of teachers implementing the activities. Prior to coding, have all your materials easily accessible, including the video, coding guide, and coding sheet. As you watch the video of the teacher implementing the activity, you will code their adherence to the activity steps and four quality indicators (i.e., modeling, scaffolding, pacing, and preparation/organization) for each activity. You will also write a short summary of the activity.

Coding guidance:

***Adherence* is about whether the teacher implements the activity steps.** The teacher should adhere to the activity directions very closely. The number of steps differs by activity, but most activities have about 10 steps. Each activity step will be coded as **1 (implemented) or 0 (not implemented)**.

***Quality* indicators focus on how well the teacher is implementing the activity.** Below is a definition and the expectations for each indicator. Each indicator will be coded as **3 (high quality), 2 (moderate quality), or 1 (low quality)**. See the table below for further coding guidance.

- **Accuracy:** This is how well the teacher models pronunciations and concepts. All modeling should be accurate. Accurate modeling includes pronouncing letter sounds correctly, not adding a schwa at the end of sounds, and smoothly blending the sounds together to read words.
- **Scaffolding:** This is how well the teacher scaffolds incorrect responses. The teacher should follow the activity directions to appropriately scaffold the child to the correct response. Each child should ultimately achieve the performance objective criterion without assistance.
- **Pacing:** This is how well the teacher uses the instructional time. The teacher should keep a brisk pace, but allow sufficient time for the children to respond. There should be little time when the children are not actively involved in the activity (i.e., time devoted to behavior management or non-instructional activities should be minimal). The activity should not seem rushed.
- **Preparation/Organization:** This is how well prepared and organized the teacher appears. The teacher should appear to be familiar with the activity format and materials. The teacher should have all materials organized and accessible when needed.

|  | **3 (High)** | **2 (Moderate)** | **1 (Low)** |
| --- | --- | --- | --- |
| **Accuracy** | Modeling is all or nearly all (≥80%) accurate. | Modeling is accurate at least half of the time. | More than half of the modeling is incorrect. |
| **Scaffolding** | All or nearly all (≥80%) errors are scaffolded. | At least half of the errors are appropriately corrected. | More than half of the errors are not appropriately corrected. |
| **Pacing** | Good pacing, with little or no down time and not rushed. | Adequate pacing, with some down time or somewhat rushed. | The pacing was too slow or too rushed. |
| **Preparation / Organization** | Well prepared and organized, with few or no lapses. | Adequately prepared and organized, but with some lapses. | Did not appear prepared or organized. |

**Summary:** Write 1-3 sentences about your overall impression of the activity. This summary may be more subjective than the other coding. Please include here anything that stood out about the activity.

- It is okay to include information that is already captured in the adherence and quality sections
  - “The teacher skipped some steps, which made it feel like she hadn’t prepared for the activity ahead of time.”
- We are *most* interested in information that is not already documented
  - “The teacher really seemed to know what she was doing, but there was one student with behavior issues and the teacher spent a lot of time redirecting that student.”
  - “The teacher added a lot of information that was not part of the activity, such as providing a definition or example for each word in the word list.”
- If there is anything you notice related to the group adaptation, please be sure to note it here.
  - “Instead of each student having their own worksheet, the teacher paired the students, and each pair had a worksheet.”
  - “The teacher wrote each word on the board rather than each student writing each word on their own.”

**Supplemental Appendix B**

Interview Guide

**Interview Guide**

1. Can you tell me a little bit about your experience with the SPELL-Links intervention?
2. How confident did you feel providing the SPELL-Links intervention to your students?
   1. What factors influenced your level of confidence to provide the intervention?
3. How did you feel about the training you received?
   1. Is there anything you would have liked more guidance on or to learn more about in the training?
   2. Was the training helpful in delivering the intervention?
   3. Did you use the SPELL-Link'd website? (Tell me more about how you used it.)
   4. Did you seek out any additional resources? (Tell me more about how you used that resource.)
4. Was the SPELL-Links intervention practical for you to implement in your classroom? (Why or why not?)
   1. Did you have any problems related to providing the intervention to your students?
   2. Were you able to implement the intervention consistently (i.e., at least four days a week)? (Why or why not?)
   3. Were you able to implement the activities in the expected amount of time (i.e., 25 minutes)? (Why or why not?)
   4. Were there particular parts of the intervention (e.g., certain activities) that were difficult or confusing to implement?
   5. Were there any parts of the intervention that you particularly liked or disliked?
5. We know the intervention was designed for one-on-one implementation, so how did you adapt the intervention activities for your small groups?
   1. Did you use the “Group Adaptation” suggestions?
6. What are some potential problems to implementing an intervention like this in a reading intervention classroom? What kinds of support might reading intervention teachers need to implement it well?
7. How effective do you think the intervention was in helping your students learn to read and write?
   1. Were there any parts of the intervention that you thought were particularly helpful or not helpful for your students?
8. Do you plan to continue to use the intervention with your students?
   1. If so, will you use the intervention as designed or will you make changes?
   2. If not, why not?
9. Do you have any other comments, suggestions, or questions?
